# Accuracy of Root ZX Electronic Apex Locator in Relation to Two Different Employment Protocols: An In Vitro Study

**DOI:** 10.7759/cureus.44659

**Published:** 2023-09-04

**Authors:** Osama S Alothmani, Amna Y Siddiqui

**Affiliations:** 1 Department of Endodontics, King Abdulaziz University, Jeddah, SAU

**Keywords:** reference length, root canal working length, root zx, employment protocol, electronic apex locator, digital measurements

## Abstract

Objective

The aim of this study is to determine the apical level of the root canal, whether it is the apical foramen or a level coronal to it, that Root ZX (J. Morita Co., Kyoto, Japan) targets and to identify its employment protocol that provides better accuracy.

Methods

Actual lengths (ALs) of 75 extracted single-rooted teeth were obtained by inserting a K-file size 8 until its tip was in level with the most coronal border of the apical foramen. Reference length (RL) was calculated by deducting 0.5 mm from AL. Roots were placed in porous sponge block soaked with Ringer’s solution, and canals were irrigated with 2 mL of 5% sodium hypochlorite. The blinded operator used Root ZX to measure lengths with K-file size 8. In the first tested employment protocol, the file was advanced to the “APEX mark” of the digital display, and the length was obtained. The second employment protocol followed the manufacturer’s recommendations by inserting the file until the “APEX mark” followed by its withdrawal to the “0.5 mark.” Stability of the digital meter for 5 seconds was mandatory before recording the lengths. All measurements were repeated one week later and then both measurements were averaged to represent “APEX mark” and “0.5 mark,” respectively. Data were analyzed using *t*-test, with significance set at 0.05.

Results

Regardless of the employment protocol, most registered lengths were longer than targeted. The mean “APEX mark” was significantly longer than the mean AL (*P*=0.000), and the mean “0.5 mark” was significantly longer than the mean RL (*P*=0.000). Although the mean “0.5 mark” was longer than the mean AL, the difference was not significant (*P*=0.07).

Conclusion

The apical level of the root canal targeted by the Root ZX was the apical foramen. The most accurate employment protocol to achieve that is to use the Root ZX according to the manufacturer’s recommendations.

## Introduction

The potential for detecting the root canal’s apical termination limit using electrical circuits was initially explored more than 100 years ago [1]. The first electronic apex locator was a single-frequency, resistance-based device [2]. Since then, many apex locators based on a variety of electrical principles have been developed [3-5]. Of these, the Root ZX (J. Morita Co., Kyoto, Japan) has been extensively tested and established itself as a standard against which new devices are compared [6]. The Root ZX calculates the impedance ratio of two simultaneously produced frequencies (0.4 and 8 kHz). This ratio decreases as the apical foramen is approached, and it has a value of 0.72 at 0.5 mm coronal to the apical foramen where the apical constriction is located [7].

Comprehensive literature review on studies evaluating the accuracy of Root ZX highlighted their variability regarding the apical reference limit used to assess the performance of the device, involving its ability to locate the apical constriction, the apical foramen, and/or the level 0.5 mm coronal to the apical foramen. The digital display mark adopted to manipulate the Root ZX also differed between studies. Several studies followed the manufacturer’s recommendations stating that the file should be extended to the “APEX mark” and then withdrawn to the “0.5 mark.” Others obtained their measurements either by extending the file to the “APEX mark” or limiting the file’s apical advancement to the “0.5 mark.” Consequently, conflicting results have been reported [3,6]. When the “0.5 mark” of the digital display was used to locate the level 0.5 mm coronal to the apical foramen, the mean range of Root ZX length was 0.16 ± 0.23 mm coronal to that level to 0.29 ± 0.32 apical to it. Range of readings within ± 0.5 mm was 74-97.4%. When the same digital display limit was used to locate the apical foramen itself, the mean Root ZX length ranged from 0.23 ± 0.39 mm coronal to the apical foramen to 0.72 ± 0.09 mm apical to it. Between 50% and 97.4% of the readings were within ± 0.5 mm from the apical foramen, while 94% to 100% of the readings were within ± 1 mm. Locating the apical foramen with the “APEX mark” yielded a mean Root ZX length range of 0.03 ± 0.22 mm coronal to the apical foramen to 0.5 ± 0.42 mm apical to it. Between 61.5% and 100% of the readings were within ± 0.5 mm from the apical foramen, with at least 97.4% of the readings within ± 1 mm [6].

The observed methodological variations in the studies and the consequent disparities in their findings could explain why many recommended using the Root ZX to locate the apical foramen [8-11], while others endorsed the manufacturer’s claim on the ability of Root ZX to accurately locate the apical constriction [7,12-14]. Precise location of the apical constriction requires histological assessment [15]. It is not clear if this approach was followed when the Root ZX was introduced [7]. Thus, it is more appropriate to consider that the Root ZX detects the level at which the impedance ratio is 0.72, which, in fact, should be 0.5 mm coronal to the apical foramen. The apical constriction may or may not be located at that level [6].

Hence, the aim of this in vitro study was to assess the accuracy of the Root ZX in locating the apical foramen and the level 0.5 mm coronal to it when used according to the manufacturer’s recommendations or when its “APEX mark” of its digital display is adopted.

## Materials and methods

A total of 75 single-rooted extracted human premolars were used. All teeth featured single canals based on bucco-lingual and mesio-distal digital periapical radiographs. Teeth were stored in 5% sodium hypochlorite (NaOCl) for 24 hours to remove any adherent organic tissue. Hand scalers were used to remove calculus. Teeth were numbered and decoronated at the level of the cemento-enamel junction to produce flat reference points, and any caries and restorations were removed. The coronal third of the root canals was enlarged using Gates-Glidden drills in sizes 4, 3, and 2 (MANI Inc, Tochigi, Japan) in a crown-down manner, and the canals were irrigated with 2 mL of 5% NaOCl. Under 4x magnification, canal patency was checked by introducing a size 8 K-file (Dentsply Maillefer, Ballaigues, Switzerland) just beyond the apical foramen. Teeth with blocked canals or broken apices were discarded and replaced. Teeth were stored in sterile saline.

**Figure 1:**
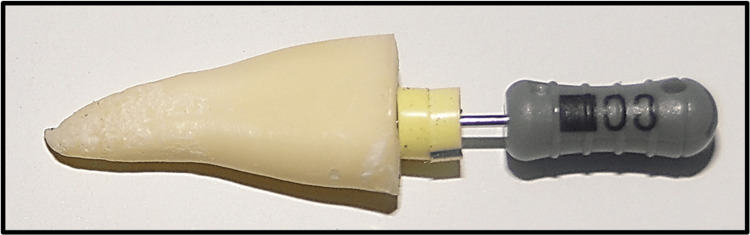
Measurement of the actual length of a decoronated premolar.

Under 4x magnification, a single operator measured the actual canal length by introducing a size 8 file with two rubber stoppers until its tip was aligned with the most coronal border of the apical foramen [8,9] (Figure 1). The stoppers were adjusted to the reference point, the file was withdrawn from the canal, and the length was measured using a caliper (Mitutoyo, Corp., Tokyo, Japan) to an accuracy of 0.01 mm. The operator obtained the first reading for the 75 teeth and then a second reading was taken. Average of the two readings represented the actual length for every canal. The reference length was obtained by subtracting 0.5 mm from the actual length.

**Figure 2:**
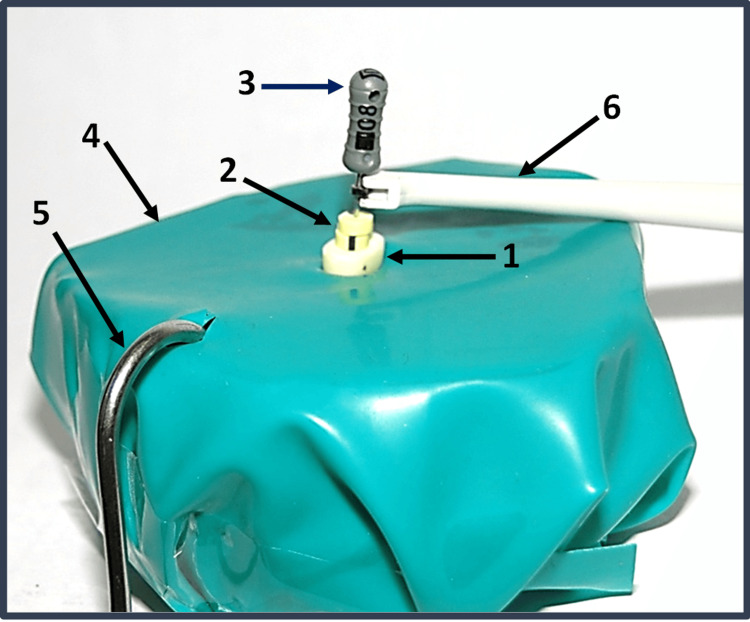
Experimental setup. 1. Decoronated premolar. 2. Double stoppers. 3. Size 8 K-file. 4. Rubber dam sheet covering a porous block soaked with Ringer’s solution. 5. Lip clip. 6. File clip.

The in vitro model used comprised plastic containers filled with a porous block (Oasis Floral Foam, Kedah, Malaysia) soaked with Ringer’s solution. A pool of solution in the base of the container ensured adequate conductivity [16]. Two small holes were made in a medium dental dam sheet (one for the tooth and one for the lip clip). The lip clip and the teeth were firmly placed in the block (one tooth at a time) with the dam stretched over the assembly (Figure 2).

The blinded operator measured the canals with Root ZX following a standard protocol. Canals were irrigated with 2 mL of 5% NaOCl delivered by a 27-gauge needle inserted as deeply as possible without engagement, and excess irrigant was removed with cotton pellets. All measurements were taken using a size 8 file with two rubber stoppers [8,9]. The first tested employment protocol comprised attaching the file to the file clip and introducing it in the canal until the display reached the “APEX mark.” If the meter gauge was stable for 5 seconds, stoppers were adjusted to the reference point, and the file was withdrawn and measured using the caliper under 4x magnification. The file was clipped back to Root ZX to test the second employment protocol and reinserted to the previous length followed by its withdrawal to the 0.5 mark of the display, which followed the manufacturer's recommendations. When the meter was stable for 5 seconds, the stoppers were adjusted to the reference point, and the length of insertion was measured as described earlier. All readings were taken twice and then averaged to represent “APEX mark” reading and “0.5 mark” readings, correspondingly. The operator determined the first set of readings for all the teeth and then the second set was obtained one week later. Data analysis using *t*-test was conducted, with level of significance set at 0.05.

## Results

Table 1 lists the mean ± standard deviation for actual length, reference length, and Root ZX lengths at the “APEX mark” and the “0.5 mark.” The mean “APEX mark” was significantly longer than the mean actual length (P = 0.000), and the mean “0.5 mark” was significantly longer than mean reference length (P = 0.000). Although the mean “0.5 mark” was longer than the mean actual length, the difference was not significant (P = 0.07). Majority of the “APEX mark” readings were longer than the actual length (Table 2), and majority of the “0.5 mark” readings were longer than the reference length and actual length. However, the “0.5 mark” registered higher accuracy in locating the apical foramen within ± 0.5 mm and ± 1 mm compared to its ability to locate the reference length (Table 3).

**Table 1:** Mean ± standard deviation (mm) for the actual length, reference length, and Root ZX lengths at the “APEX mark” and the “0.5 mark”

Measurement	Mean ± standard deviation (mm)
Actual length	13.32 ± 1.34
Reference length	12.82 ± 1.33
APEX mark	14.27 ± 1.49
0.5 mark	13.74 ± 1.45

**Table 2:** Frequency distribution of Root ZX measurements at the “APEX mark” in relation to the actual length (%)

Root ZX measurement		Compared to the actual length
APEX mark (%)	Shorter	4.0
	Equal	1.33
	Longer	94.67
	Within ± 0.5 mm	17.0
	Within ± 1 mm	57.0

**Table 3:** Frequency distribution of Root ZX measurements at the “0.5 mark” in relation to reference and actual lengths (%)

Root ZX measurement		Compared to the reference length	Compared to the actual length
0.5 mark (%)	Shorter	4.0	16.0
	Equal	1.33	4.0
	Longer	94.67	80.0
	Within ± 0.5 mm	19.0	53.0
	Within ± 1 mm	58.0	86.0

## Discussion

The objective of this in vitro investigation was to determine whether the Root ZX should be used to locate the apical foramen or to detect the level coronal to the apical foramen by 0.5 mm. Furthermore, we intended to establish which employment protocol would allow for higher precision when using Root ZX. Results showed that Root ZX should be used following the manufacturer’s recommendation to preferably locate the apical foramen instead of locating the level coronal to the apical foramen by 0.5 mm (Tables 1, 3). Adopting the “APEX mark” of the digital display led to frequently establishing the file tip far beyond the apical foramen (Tables 1, 2).

The results of the current study were in line with previous studies recommending the use of Root ZX to locate the apical foramen [8-11], and that the best approach to achieve this was using the Root ZX in accordance to the manufacturer’s recommendations as 86% of theses readings were within ± 1 mm from the apical foramen (Table 3). The lenient tolerance limit of ± 1 mm provides a clinically suitable range to report the accuracy of electronic apex locators [17,18].

Coronal pre-flaring of the canals was done before using Root ZX because this step improves the device’s accuracy [19-21]. In many teeth, the apical foramen was inclined in a bucco-lingual direction. Thus, the most coronal border of the apical foramen was chosen as a guide to standardize measurements. The use of double stoppers was intended to reduce potential errors from movement while measuring file length [8,9,17]. All measurements were taken twice [9,22,23]. Following this protocol, a total of 450 readings were obtained: 150 recordings to determine the actual length and 300 measurements acquired by the Root ZX. In order to avoid bias, the operators obtained the first set of readings for the entire sample and then went through them again to obtain the second set instead of obtaining both readings for each tooth consecutively. This was done to reduce the chance of remembering the recorded length. Furthermore, the operator who used Root ZX was blinded to the reference lengths. We used the Root ZX with K-file size 8 since this size provided significantly higher accuracy compared to size 15 [9]. The use of 5% NaOCl should not have affected our results since the accuracy of Root ZX was not affected by the different concentrations of NaOCl [22,24]. Overall, rigorous efforts were implemented to standardize and control potential factors that could impact the results of the current study. Yet, the reported accuracy of Root ZX was lower than previously stated [6].

The in vitro model used in the current study has been used only once before and showed that using Tri Auto ZX established the file tip coronal to the apical foramen by a mean length of 0.78 ± 0.47 mm, while using Raypex 5 combined with Endo IT motor established the file tip coronal to the apical foramen by a mean length of 0.66 ± 0.44 mm. Neither device led to violation of the apical foramen [16]. Such results differ from the findings of the current study and could be attributed to the inherent differences between the operation mode of Root ZX compared to controlling the length while mechanically enlarging the canals. Besides using rotary files, which have larger size and taper compared to hand files, the latter approach leads to auto-reversing the file once it reaches a certain limit instead of identifying a precise working length. Hence, the possibility of violating the apical foramen is minimized. The extent to which the type of the embedding medium has influenced our results is unknown. Circuits of electronic apex locators might be subjected to different and multiple factors affecting their readings in vivo. Thus, the extent to which in vitro findings could be clinically applicable is undetermined [25]. Nevertheless, in vitro/ex vivo assessments of electronic apex locators have been validated and considered comparable to in vivo evaluations [26-28].

A high frequency of readings longer than targeted has been observed in the present study (Tables 2, 3). Deciding the final working length might differ from case to case consequent to the possible need for adjustment of the length indicated by the electronic apex locators. Confirming the length specified by the electronic apex locator with a periapical radiograph decreased the frequency of over-instrumentation [17,29]. Another approach to reduce the chance for over-instrumentation is to enlarge the canals to a level shorter than (coronal to) the length indicated by the electronic apex locator by 0.5 to 1 mm [30]. Further studies are needed to verify protocols to reduce the potential for frequent long readings recorded by electronic apex locators. Such studies could compare the accuracy of electronic working length determination with the length obtained by preoperative cone-beam computed tomography images.

Potential limitation of this study could be that it was conducted in vitro. Despite our laborious efforts to replicate clinical situations while obtaining Root ZX measurements, it is still possible that certain unaccountable factors under in vivo conditions might lead to altered precision. Hence, the results of this study should be cautiously extrapolated to the clinical setting, and every effort should be taken to accurately determine the working length. Furthermore, the results obtained might vary if another electronic apex locator was used as these devices differ in their electrical circuits and principles.

## Conclusions

Within the limitations of this study, the Root ZX targeted the apical foramen. This was best attained when it was used according to the manufacturer’s recommendations. Additional measures to prevent the chance for possible over-instrumentation should be undertaken.
